# Case Report on Rare Presentation of Intravenous Leiomyomas with Intracardiac Leiomyomatosis

**DOI:** 10.5070/M5.52111

**Published:** 2026-10-31

**Authors:** Star Q Lopez, Natalie Llauro, Anvesh Macherla, Lindsey Spiegelman, Roozbeh Houshyar

**Affiliations:** * University of California, Irvine, School of Medicine, Irvine, CA; ^ Weill Cornell Medicine, Department of Anesthesiology, New York, NY; † University of California, Irvine, Department of Emergency Medicine, Orange, CA; ** University of California, Irvine, Department of Radiology, Orange, CA

## Abstract

Uterine leiomyomas, or fibroids, are the most prevalent benign tumor pathology affecting women.^1^ Fibroids are reportedly found in nearly 70% of Caucasian women and more than 80% of women with African ancestry.^1^ Rarely, fibroids progress to uterine intravenous leiomyomas (IVL), a neoplastic growth pattern characterized by intravascular proliferation of this smooth muscle cell tumor.^2^ This tumor is typically confined to the pelvic vasculature but can occasionally spread with venous blood flowing to the heart, ultimately penetrating the inferior vena cava, right heart chambers, and pulmonary arteries.^3^ If the tumor reaches the heart, this intracardiac leiomyomatosis (ICL) can result in severe cardiac symptoms including arrythmias, murmurs, and sudden death.^3^,^4^ The case presentation shows both IVL and ICL in a patient.

Approximately 12 months following hysterectomy due to fibroids, the patient presented with mild dyspnea on exertion, retrosternal chest pain, and a syncopal episode. She was diagnosed with saddle embolism with extension into right ventricle and complete occlusion of the inferior vena cava (IVC). Informed by imaging from echocardiogram, computed tomography (CT) angiogram of the chest, CT of the abdomen and pelvis with contrast and magnetic resonance imaging (MRI) of the abdomen and pelvis, providers proceeded with mechanical thrombectomy followed by IVC filter, tumor debulking surgery, resection of a pelvic mass, and proximal vaginectomy. To facilitate early detection and treatment, providers should factor the risk of IVL and ICL into ongoing care and patient education post hysterectomy or fibroid diagnosis.

**Topics:**

Uterine leiomyoma, fibroid, benign tumor, intravenous leiomyoma, intracardiac leiomyomatosis, neoplastic growth, pelvic vasculature, pulmonary arteries, inferior vena cava, arrhythmia, murmur, sudden death, hysterectomy, syncope, pulmonary embolism, saddle embolism, CT angiogram chest, CTA, MRI, thrombectomy, CT abdomen pelvis, MRI.

**Figure:**
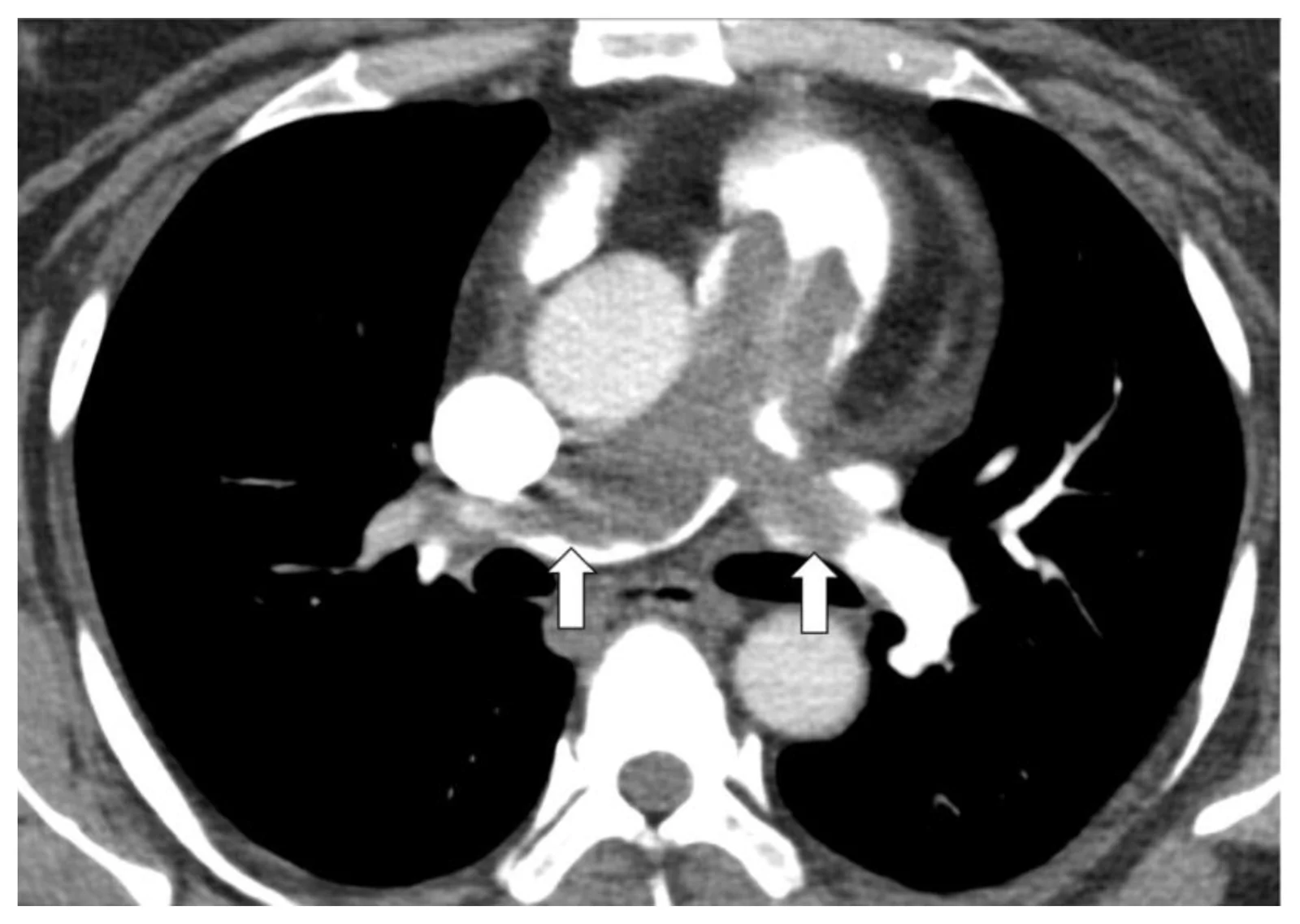


**Figure:**
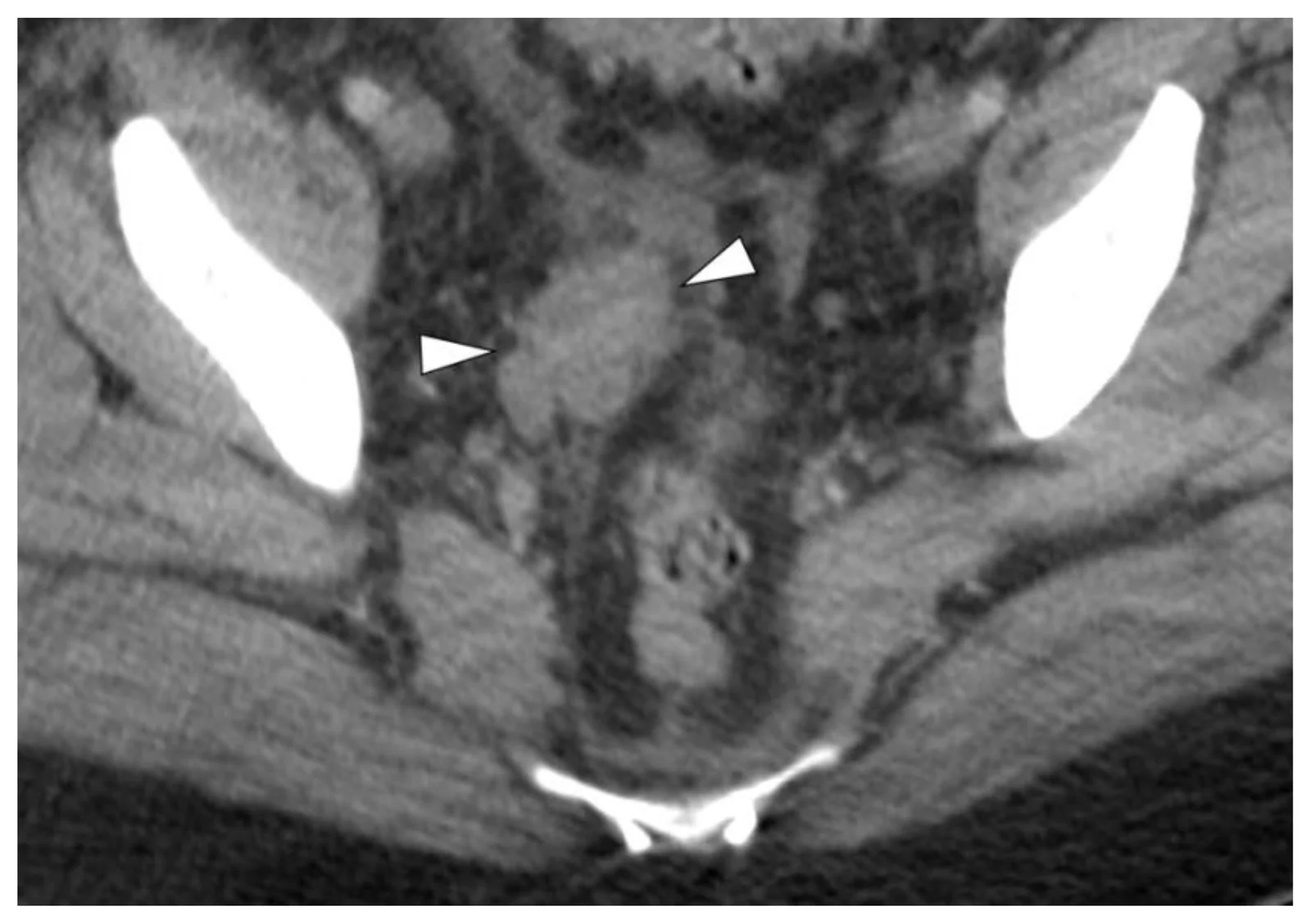


**Figure:**
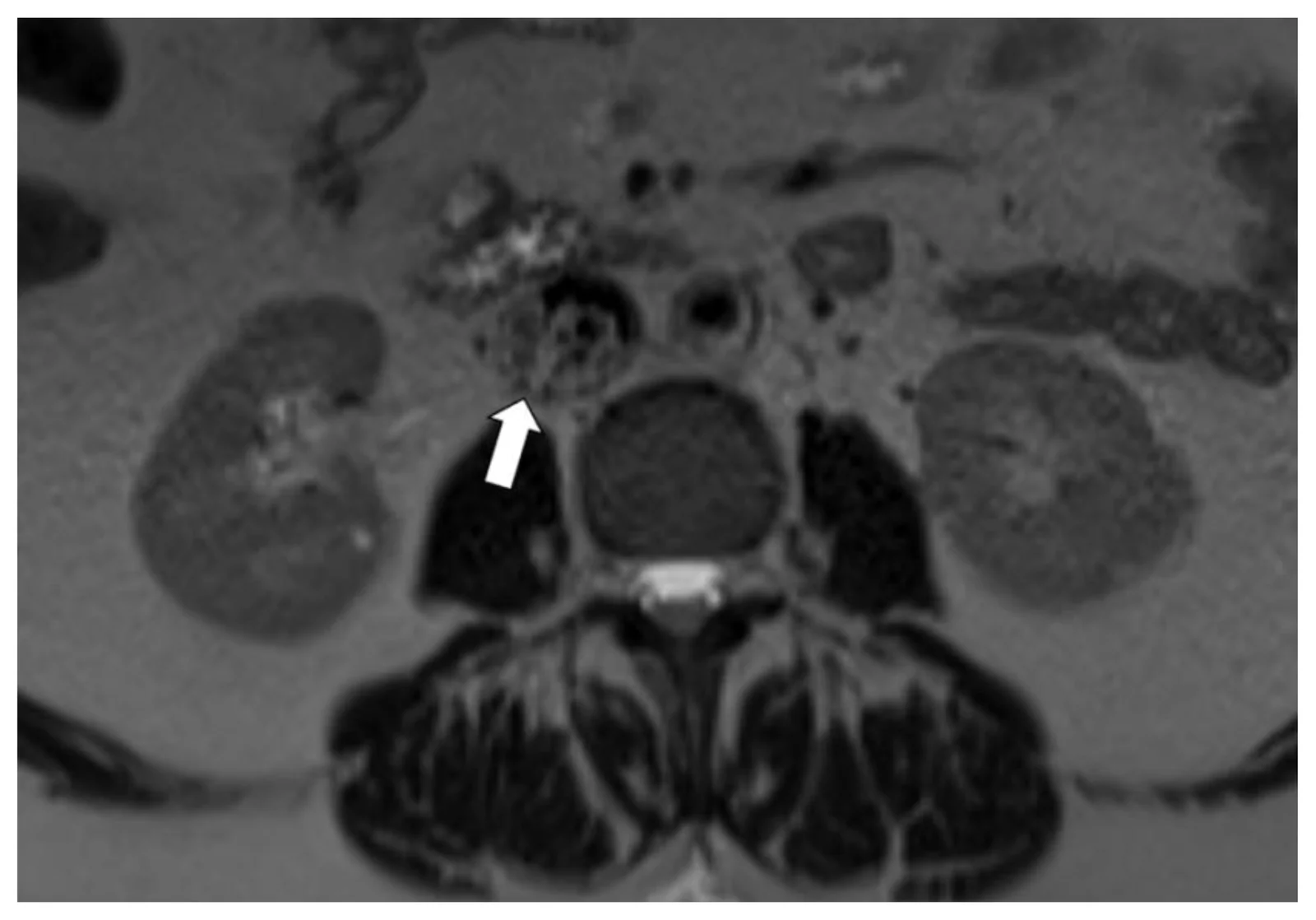


**Figure:**
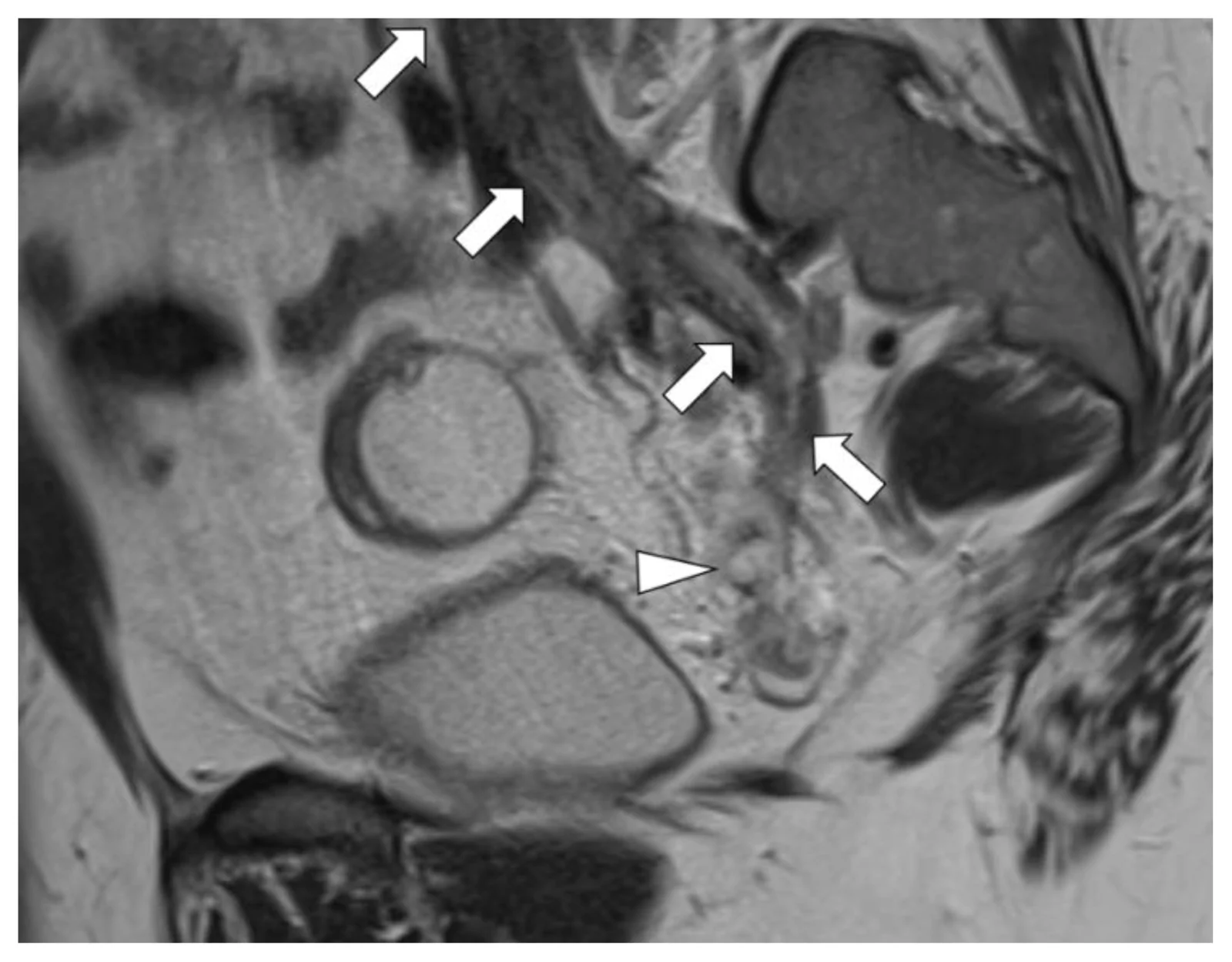


## Brief introduction

A patient with a history of uterine leiomyomas and surgical history of hysterectomy presented to an outside hospital with mild dyspnea on exertion, retrosternal chest pain, and syncopal episode including 5 minutes of unconsciousness. Imaging revealed bilateral pulmonary emboli with right heart strain, clotting in both the right atrium and ventricle, and complete occlusion of the IVC, requiring mechanical thrombectomy. Later imaging showed lobulated tissue masses in the right hemipelvis and superior right vaginal cuff. Moreover, imaging demonstrated filling defects consistent with thrombosis. Ultimately, the patient exhibited both IVL and ICL, requiring multiple surgeries and continued care.

## Presenting concerns and clinical findings

A 39-year-old female presented to an outside hospital’s emergency department after a syncopal episode. She woke up to urinate, felt short of breath while on the toilet, and subsequently lost consciousness for five minutes. Prior to her visit, she reported one month of new retrosternal chest pain and shortness of breath with exertion. On evaluation, she was found to be tachypneic and dyspneic with the following relevant lab work: a white blood cell count of 14 k/uL (4.3 – 10.0 k/uL), D-dimer of 2.44 ug/mL (0.00 – 0.50 ug/mL FEU), international normalized ratio (INR) of 1.2 (0.8 – 1.2), and troponin of 0.392ng/mL (0.000 – 0.040 ng/mL), Her electrocardiogram (ECG) showed a regular rate, sinus rhythm, no evidence of axis deviation, and low voltage incomplete right bundle branch block (RBBB) with nonspecific ST-T segment changes.

Approximately 12 months prior to her presentation, her past surgical history was significant for a hysterectomy due to uterine fibroids and menorrhagia. The patient’s medical history is significant for primary hypertension, fatty liver, heart murmur, and diabetes.

## Significant findings

During emergency room evaluation, the patient’s CT angiogram of her chest revealed bilateral pulmonary embolism, saddle embolism (arrows) and clot extension into right atrium, right ventricle, complete occlusion of IVC, a 4 mm pulmonary nodule within the posterior aspect of the right upper lobe, and a 5 mm nodule in the posterior aspect of the right lower lobe.

The patient was immediately taken to the operating room by cardiothoracic surgery for an open mechanical thrombectomy. Intraoperatively, surgeons found a very large, well organized chronic thrombus with cystic components adherent to the patient’s IVC. The components extended across the right atrium to the tricuspid valve into the right ventricle and into both left and right main pulmonary arteries. Postoperatively, patient was admitted to the ICU and placed on a heparin drip. She was ultimately discharged on apixaban. A CT angiogram of the chest postoperatively showed complete eradication of intracardiac thrombus.

Postoperatively, a CT of the abdomen and pelvis with contrast showed a lobulated soft tissue density (arrowheads) measuring 4.5 × 3 cm in the right pelvis. A subsequent MRI of the pelvis showed a 3.7 × 2.8 cm lobulated structure in the right pelvis above the vaginal cuff thought to be most consistent with an unusual cluster of venous structures. Following this, a CT venogram of the abdomen and pelvis revealed residual filling defects in the infrarenal IVC, extending through the right common iliac vein and multiple internal iliac branches.

Patient saw gynecologic oncology and vascular surgery teams as an outpatient. Further imaging was obtained at this time. The patient’s CT of the abdomen and pelvis with contrast demonstrated a 3.8 × 3.2 cm lobulated soft tissue mass in the right hemipelvis that was deemed consistent with residual leiomyoma from her prior resection. This is in continuity with a hyperexpanded right internal iliac vein with extension into the IVC, which was suspicious for the patient’s known IVL. Further MRI imaging revealed a lobular enhancing mass measuring 4.8 × 3.8 cm at the superior right vaginal cuff. A sagittal T2 weighted MRI of the pelvis demonstrated invasion of the right internal iliac vein and IVC (arrows) from the residual right leiomyoma (arrowhead). An axial T2 weighted image of the abdomen showed a T2 intermediate mass (arrow) in the IVC consistent with intravascular invasive leiomyomatosis. There was also enlargement of and filling defects within the right internal iliac and common iliac veins and infrarenal inferior vena cava, suggesting thrombosis.

## Patient course

To prevent any clot burden traveling to the heart, vascular surgery placed an IVC filter. Following this, the patient underwent an exploratory laparotomy, tumor debulking, bilateral salpingo-oophorectomy, resection of the right internal iliac vein, and IVC venotomy with open thrombectomy of tumor with the gynecologic oncology and vascular surgery teams. She was admitted to the surgical ICU postoperatively under the gynecologic oncology team.

Several months later, the patient was re-imaged with a CT of the chest with contrast, revealing pulmonary metastasis, specifically a right upper lobe pulmonary nodule that had increased from 4 mm to 6 mm in size. At that time, a CT of the abdomen and pelvis with contrast found the right vaginal cuff mass measured 4.3 × 2.6 cm. Patient subsequently underwent surgical resection of the pelvic mass and proximal vaginectomy. The patient was discharged in stable condition post operatively and continues to be followed by gynecologic oncology.

## Discussion

Intravenous leiomyomatosis is characterized as a rare, benign smooth muscle tumor found within veins.^5^ It is an aggressive tumor with reported cases showing invasion to the IVC (93.1%), right atrium (87.4%) and ventricle (47.1%) of the heart.^6^ Common primary sites of origin are parauterine vessels, broad ligament vessels, and uterine veins.^7^,^8^ The tumor most commonly spreads via the internal iliac vein system accounting for approximately 71.4% of cases.^10^ In this pathway, growth typically begins in the parauterine vessels and progresses through the iliac veins before extending into the inferior vena cava.^8^,^9^ Less frequently, extension occurs via the ovarian vein, representing 14.3% of cases, while an additional 14.3% of cases demonstrate involvement of both pathways.^9^

From an emergency medicine perspective, IVL is clinically important because patients often present with nonspecific cardiopulmonary symptoms that overlap with more common and life-threatening diagnoses. Patients with IVL typically present with cardiac symptoms such as dyspnea upon exertion, chest pain, and syncopal episodes, but some patients may have more severe symptoms such as pulmonary embolism and sudden death.^4^,^6^ These symptoms are due to the venous obstruction caused by the tumor.^4^ Patients may also present with abnormal vaginal bleeding, pelvic pain, abdominal pain, and engorgement of lower extremities.^3^,^5^

Only female patients are at risk for development of IVL and in many cases, it is seen in those that have previously undergone a hysterectomy.^4^ As a result, IVL should be considered in the differential diagnosis of female patients presenting with unexplained right-sided cardiac masses, venous obstruction, or pulmonary embolism-like symptoms, particularly in those with a history of a prior hysterectomy.^10^,^11^ In a review of 31 IVL cases, it was shown that 64% of female patients with IVL had previously undergone a hysterectomy.^4^ IVL is more common in premenopausal white women with previous pregnancies.^4^,^5^ This is reflective of our case, where the patient underwent a hysterectomy for uterine fibroids 12 months prior to admission for IVL. For emergency clinicians, this highlights the importance of obtaining a thorough gynecologic and surgical history when evaluating unexplained cardiopulmonary symptoms in female patients.

Two primary theories have been proposed regarding the development of IVL. One theory suggests IVL arises directly from the vein walls while the other hypothesizes IVL arises from vascular invasion of the myometrium by a uterine leiomyoma.^4^,^5^

Multiple different imaging modalities can be useful in the detection and diagnosis of IVL, but the most sensitive methods for diagnosis include MRI and CT with contrast.^5^. Computed tomography may prove more clinically useful due to CT’s relatively enhanced spatial resolution, fewer artifacts, time-efficiency, and inclusion of patients with cardiac pacemakers and/or metal intrauterine devices.^12^,^13^ On contract-enhanced CT, IVL appears as a heterogenous enhancement with lower density than the enhanced venous blood.^9^ The characteristic imaging patterns include the “luffa sponge” appearance on coronal and sagittal reconstructions and “sieve” appearance on axial imaging.^12^ When there is right heart involvement, the lesions display a “walking stick head” or “snake head” appearance on coronal and sagittal images.^12^ For MRI, there is a “luffa sponge” appearance on coronal Fast Imaging Employing Steady-State Acquisition (FIESTA) images and a “sieve pore” appearance on T2-weighted axial images.^14^ The tumor typically shows intermediate signal intensity on T1-weighted images and heterogenous high signal on T2-weighted sequences.^15^,^16^ Ultrasound can be particularly useful for real-time visualization of the tumor and typically shows “sieve hole” and “multi-track” signs.^12^,^17^ On echocardiography, IVL appears as a highly mobile sessile mass in the right atrium with moderate-to-low echogenicity originating from the IVC.^8^

The primary treatment for IVL is total surgical excision.^18^ The rate of IVL recurrence is 16–33%; thus it is vital that the tumor is completely removed to avoid recurrence.^18^,^19^ Surgical excision may be done in a one-stage approach involving tumor removal via a transabdominal surgery or a thoracoabdominal two-stage approach.^3^,^18^ A one-stage approach is favored if IVL has not adhered to vessel walls and has not affected the right heart.^18^

Depending on the severity of IVL, additional procedures, such as tricuspid valve replacement, tricuspid valve annuloplasty, or aortocoronary bypass grafting, may be indicated.^5^ This patient underwent several procedures to treat her tumors including open mechanical thrombectomy followed by tumor debulking surgery. After identification of concerning growth in a right vaginal cuff mass, the patient underwent pelvic mass resection and vaginectomy.

Additionally, hormone therapies may play a role in the treatment of IVL. These tumors express estrogen and progesterone receptors and tumor growth may be influenced by hormonal levels.^19^ Thus, it is important to avoid exogenous estrogen and thereby reduce the growth of any additional leiomyomas.^20^ As of now, the efficacy of gonadotropin-releasing hormone agonists, aromatase inhibitors, and selective estrogen receptor modulators has not been demonstrated.^19^,^20^ However, in some cases, gonadotropin-releasing hormone agonists have been utilized to prevent tumor growth and recurrence in unresected IVL.^19^,^20^

While definitive treatment is surgical, the role of the emergency medicine provider is early recognition, stabilization, and appropriate disposition. Given the prevalence of fibroid diagnoses among women and the common surgical history of hysterectomy among the majority of IVL patients, providers should stay alert to the potential risk associated with said population when approaching patient care and education. Both CT and MRI afford providers and patients valuable information to afford life-saving measures and prevent life-threatening outcomes.

Ultimately, early identification of IVL in the emergency department is critical because delayed diagnosis can lead to significant morbidity due to cardiac obstruction or embolic complications. Maintaining a broad differential and recognizing key historical and imaging features can facilitate timely diagnosis and coordination of care, improving patient outcomes.^21^,^22^

## Supplementary Information





















## References

[1] Baird DD, Dunson DB, Hill MC, Cousins D, Schectman JM (2003). High cumulative incidence of uterine leiomyoma in black and white women: Ultrasound evidence. Am J Obstet Gynecol.

[2] Wang HC, Wang YB, Chen XH, Cui LL (2016). Uterine intravenous leiomyomatosis with intracardiac extension and pulmonary benign metastases on FDG PET/CT: A case report. Korean J Radiol.

[3] Lou YF, Shi XP, Song ZZ (2011). Intravenous leiomyomatosis of the uterus with extension to the right heart. Cardiovasc Ultrasound.

[4] Harris LM, Karakousis CP (2000). Intravenous leiomyomatosis with cardiac extension: Tumor thrombectomy through an abdominal approach. J Vasc Surg.

[5] Nam MS, Jeon MJ, Kim YT, Kim JW, Park KH, Hong YS (2003). Pelvic leiomyomatosis with intracaval and intracardiac extension: A case report and review of the literature. Gynecol Oncol.

[6] Matsuo K, Fleischman F, Ghattas CS (2012). Successful extraction of cardiac-extending intravenous leiomyomatosis through gonadal vein. Fertil Steril.

[7] Jalaguier-Coudray A, Allain-Nicolai A, Thomassin-Piana J (2017). Radio-surgical and pathologic correlations of pelvic intravenous leiomyomatosis. Abdom Radiol (NY).

[8] Wang Q, Li Y, Gu X (2023). Imaging diagnosis of intravenous leiomyomatosis: An institutional experience. Clin Radiol.

[9] Ma H, Niu Y, Yang Z, Zheng M (2022). Echocardiographic characteristics and contrast-enhanced imaging of intravenous leiomyomatosis with intracardiac extension. J Ultrasound Med.

[10] Kloska M, Patel P, Soliman A, Musco K, Rovella J (2021). Intravenous leiomyomatosis: An uncommon cause of pulmonary embolism. Am J Case Rep.

[11] Han Y, Chung YJ, Shin I (2021). Intravenous leiomyomatosis misdiagnosed with large thrombosis in inferior vena cava. Taiwan J Obstet Gynecol.

[12] Wang H, Nie P, Chen B (2018). Contrast-enhanced CT findings of intravenous leiomyomatosis. Clin Radiol.

[13] Ge Z, Wang Y, Qi Z, Zhang Q, Jin J, Li J (2019). Ultrasound appearance of intravenous leiomyomatosis: A case report. Medicine (Baltimore).

[14] Gui T, Qian Q, Cao D, Yang J, Peng P, Shen K (2016). Computerized tomography angiography in preoperative assessment of intravenous leiomyomatosis extending to inferior vena cava and heart. BMC Cancer.

[15] Kang LQ, Zhang B, Liu BG, Liu FH (2012). Diagnosis of intravenous leiomyomatosis extending to heart with emphasis on magnetic resonance imaging. Chin Med J (Engl).

[16] Zeng H, Xu Z, Zhang L (2016). Intravenous leiomyomatosis with intracardiac extension depicted on computed tomography and magnetic resonance imaging scans: A report of two cases and a review of the literature. Oncol Lett.

[17] Ge Z, Wang Y, Wang Y (2023). Diagnostic experience of intravenous leiomyomatosis with emphasis on conventional ultrasonography imaging: A single-center study. Front Oncol.

[18] Luo G, Pan H, Bi J (2019). Surgical treatment of intravenous leiomyomatosis involving the right heart: A case series. J Int Med Res.

[19] Mathey MP, Duc C, Huber D (2021). Intravenous leiomyomatosis: Case series and review of the literature. Int J Surg Case Rep.

[20] Low HY, Zhao Y, Huang KS, Shen HP, Wu PJ, Tseng CJ (2017). Intravenous leiomyomatosis of the uterus: A clinicopathological analysis of nine cases and literature review. Taiwan J Obstet Gynecol.

[21] Liu J, Liang M, Ma G (2018). Surgical treatment for intravenous-cardiac leiomyomatosis. Eur J Cardiothorac Surg.

[22] Aleksandrov A, Lyubenov A, Damyanova P (2023). Intravascular leiomyomatosis with cardiac and pelvic involvement in a postmenopausal woman: A case report of multidisciplinary team management. Case Rep Womens Health.

